# MRI-based machine learning analysis of perivascular spaces and their link to sleep disturbances, dementia, and mental distress in young adults with long-time mobile phone use

**DOI:** 10.3389/fnins.2025.1555054

**Published:** 2025-04-28

**Authors:** Li Li, Yalan Wu, Jiaojiao Wu, Bin Li, Rui Hua, Feng Shi, Lizhou Chen, Yeke Wu

**Affiliations:** ^1^Department of Radiology, Hospital of Chengdu University of Traditional Chinese Medicine, Chengdu, Sichuan, China; ^2^Department of Research and Development, United Imaging Intelligence, Shanghai, China; ^3^Department of Radiology, West China Hospital of Sichuan University, Chengdu, Sichuan, China; ^4^Department of Stomatology, Hospital of Chengdu University of Traditional Chinese Medicine, Chengdu, China

**Keywords:** mobile phone use, computational MRI-visible EPVSs, sleep disturbances, dementia, mental distress, young adults

## Abstract

**Objective:**

Long-term mobile phone use (LTMPU) has been linked to sleep disorders, mood disorders, and cognitive impairment, with MRI-detected enlarged perivascular spaces (EPVSs) as potential imaging markers. This study investigated computational MRI-visible EPVSs and their association with sleep disturbance, dementia, and mental distress in young adults with LTMPU.

**Methods:**

This retrospective study included 82 LTMPU patients who underwent MRI scans and assessments using six clinical scales: Montreal Cognitive Assessment (MoCA), Pittsburgh Sleep Quality Index (PSQI), Insomnia Severity Index (ISI), Epworth Sleepiness Scale (ESS), Hamilton Anxiety (HAM-A), and Hamilton Depression (HAM-D). Deep learning algorithms segmented EPVSs lesions, extracting quantitative metrics (count, volume, mean length, and mean curvature) across 17 brain subregions. Correlation analyses explored relationships between EPVSs indicators and clinical measurements. The BrainNet Viewer tool highlighted significant brain subregions and EPVSs traits linked to dementia, sleep disturbance, and mental distress.

**Results:**

Correlation analyses identified 23 significant indicator pairs (FDR-adjusted *p* < 0.05), including associations between nine EPVSs characteristics and MoCA scores: four with the PSQI, one with the ISI, three with the ESS, four with the HAM-A, and two with the HAM-D. Regression analyses revealed seven significant EPVSs features, with three linked to cognitive impairment: mean EPVSs length in the left basal ganglia and mean length/curvature in the left temporal lobe. Also, the mean EPVSs length in the left frontal lobe could indicate insomnia, sleepiness, and anxiety.

**Conclusion:**

Computational EPVSs metrics offer insights into the EPVSs pathophysiology and its links to mood disorders, sleep disturbances, and cognitive impairment in LTMPU patients. These findings also highlight potential connections between EPVSs, excessive daytime sleepiness, and anxiety, contributing to a comprehensive understanding of these multifaceted conditions.

## 1 Introduction

The number of mobile phones has markedly increased in recent decades. The number of mobile Internet users in China was 1.029 billion, with young adults making up a significantly high proportion of such users (19.7%), as of December 2021 (Zhang et al., 2023). Long-time mobile phone use (LTMPU) is defined as engaging with a mobile device ≥ 4 h/day (Liu et al., 2019). Recognition of the negative effects of mobile phone overuse is increasing. Previous research has highlighted the correlation between excessive mobile phone usage and a spectrum of health issues, including sleep disturbances, excessive daytime sleepiness (EDS), poor sleep quality, dementia, anxiety, depressive disorders, and so on (Abi-Jaoude et al., 2020; Li et al., 2020; Liu et al., 2019; Nowak et al., 2022; Wang et al., 2024a; Zhao et al., 2023).

Sleep disturbances (particularly insomnia disorders, EDS, and poor sleep quality) frequently co-occur with various medical and psychiatric conditions that inherently affect sleep, including dementia and mood disorders (anxiety and depressive disorders) (McCarter et al., 2022; Pérez-Carbonell et al., 2022; Sutton, 2021). Our understanding of the precise neurobiology and neural underpinnings of insomnia disorders, EDS, subjective sleep quality, mood disorders, and dementia remains limited (Aquino et al., 2024; Chouliaras and O'Brien, 2023; Craske and Stein, 2016; Malhi and Mann, 2018; McCarter et al., 2022; Pérez-Carbonell et al., 2022). In practice, conditions such as insomnia disorders, EDS, subjective sleep quality, dementia, anxiety disorders, and depressive disorders often pose challenges to clinicians because of the complexities involved in their detection, diagnosis, and variability in treatment responses (Perlis et al., 2022; Pérez-Carbonell et al., 2022; McCarter et al., 2022; Wang et al., 2024b; Chellappa and Aeschbach, 2022; Blumberger et al., 2018). It is imperative to unravel their mechanisms to prevent or defer the onset of these conditions and mitigate progression.

Evidence suggests that perivascular spaces (PVSs) dysfunction is involved in the pathogenesis of sleep disturbances, Alzheimer’s disease, and other neurodegenerative and inflammatory disorders (Rasmussen et al., 2022; Wardlaw et al., 2020). PVSs include a variety of passageways around the arterioles, capillaries, and venules in the brain and play an essential role in forming a network of drainage channels and the glymphatic system for eliminating metabolic waste and fluid from the brain (Ding et al., 2017; Wardlaw et al., 2020). Sleep is crucial for brain clearance in humans, and one night of sleep deprivation leads to reduced clearance of intrathecally delivered MRI contrast agents from the brain parenchyma across multiple brain regions (Rasmussen et al., 2022). PVSs dysfunction serves as a marker of neuroinflammation (Fang et al., 2020), and is associated with mood disorders (anxiety and depressive disorders) (Guo et al., 2023). Previous studies have indicated that glymphatic inhibition resulting from sleep disturbances could be a common pathway leading to cognitive impairment in the elderly and promoting the progression of neurodegenerative disorders (Rasmussen et al., 2022). The glymphatic system, with PVSs as a key component, plays a crucial role in the clearance of brain amyloid β (Aβ) and tauopathy, which are linked to neurodegenerative conditions (cognitive impairment) (Wang et al., 2022).

MRI is a pivotal tool for the diagnosis and prognosis of various neuropsychiatric disorders, including sleep disturbances, dementia, and depressive or anxiety disorders, and for monitoring treatment progress. MRI-visible enlarged perivascular spaces (EPVSs) visualized on structural MRI scans, mark perivascular space dysfunction, impairment of normal brain fluid, waste clearance and microvascular dysfunction, and impaired glymphatic exchange (Rocca et al., 2023; Wardlaw et al., 2020). Previous studies on epilepsy, long-term COVID-19, cerebral small vessel disease, and post-stroke have indicated an association between MRI-visible EPVSs and insomnia disorder (Sotgiu et al., 2023), subjective poor sleep quality (Del Brutto et al., 2022), dementia (Ding et al., 2017), and depressive disorders (Liang et al., 2018), while studies exploring the association between MRI-visible EPVSs and anxiety and EDS are limited.

Currently, a growing body of research is focused on the computational quantification of EPVSs, facilitated by advancements in the analysis of extensive datasets in which visual rating scores and scales are either absent or deemed unreliable for assessing EPVSs in the centrum semiovale (Del Brutto et al., 2022; Waymont et al., 2024). Recent advancements in automated segmentation algorithms, such as the VB-Net architecture, have enabled high-precision volumetric and morphological analyses of EPVSs, addressing the inconsistencies inherent in manual grading methods. In our previous work, VB-Net achieved a recall and precision of 0.953 and 0.923, respectively, demonstrating exceptional reliability in EPVSs quantification (Zhang et al., 2024). This framework combines an encoder-decoder design for feature embedding, residual connections for stable gradient propagation, and bottleneck layers for computational optimisation, as detailed in previous publications (Zhu et al., 2022).

In this study, we aimed to demonstrate the features of computational MRI-visible EPVSs and their association with sleep disturbances, dementia, and mental distress in young adults with LTMPU.

## 2 Materials and methods

### 2.1 Participants

This school-based, cross-sectional study was conducted between October 2021 and May 2022. A total of 165 students and young teachers aged 18–50 years at a medical college in the Wenjiang District, Chengdu, China, were recruited. Of these, 146 (88.5%) were valid. Questionnaires were distributed to the students and young teachers during class. The Ethics Committee of the Hospital of Chengdu University of Traditional Chinese Medicine approved this study.

The inclusion criteria were as follows: (a) LTMPU. The duration of mobile phone use per day was determined using the following question: How long do you usually spend using mobile phones per day? The response categories for this question were less than 2 h, 2–4 h, 4–6 h, and more than 6 h. LTMPU was defined as using a mobile phone ≥ 4 h per day in consideration of the recent findings (Liu et al., 2019); (b) ethnic Han; (c) being free of any psychoactive medication at least 2 weeks before and during the study (Li et al., 2016); (d) right-handedness assessed with the Edinburgh Handedness Inventory (Oldfield, 1971). Exclusion criteria were as follows: (a) with coronavirus disease 2019 (COVID-19) infections; (b) with any significant neuropsychiatric disease or brain structural abnormality; (c) with MRI contraindications.

To evaluate mental status, cognitive function, and sleep status, all participants were asked to complete the Hamilton Anxiety (HAM-A), Hamilton Depression (HAM-D), Montreal Cognitive Assessment (MoCA), Epworth Sleepiness Scale (ESS), Pittsburgh Sleep Quality Index (PSQI), and Insomnia Severity Index (ISI). The HAM-A was used to assess the severity of anxiety symptoms. The total comprehensive HAM-A scores ranged from 0 to 56. A HAM-A score ≤ 7 indicates *no or minimal anxiety*; 8–14 indicates *mild anxiety*; 15–23 indicates *moderate anxiety*; and ≥ 24 indicates *severe anxiety* (Chen et al., 2020; Matza et al., 2010). The severity of depressive symptoms was assessed using the Hamilton Depression Rating Scale. A global HAM-D score ranged from 0 to 54. A HAM-D score ≤ 7 indicates no depression; 8–16 indicates mild depression; 17–23 indicates moderate depression; and ≥ 24 indicates *severe depression* (Zimmerman et al., 2013).

The severity of cognitive impairment was assessed using the MoCA. The total MoCA score ranged from 0 to 30. Cognitive impairment is defined as a score < 26. The lower the MoCA score, the worse the cognitive function (Chen et al., 2020). The severity of excessive daytime sleepiness symptoms was assessed using the ESS. The total ESS scores ranged from 0 to 24. An ESS score of more than 6, 11, and 16 was defined as sleepiness, excessive sleepiness, and risky sleepiness, respectively (Hou et al., 2022). The severity of subjective sleep quality was assessed using the PSQI. The total PSQI score ranged from 0 to 21. A score > 5 indicated *poor sleep quality* (Morin et al., 2011). The severity of insomnia was assessed using the ISI. The total ISI score ranged from 0 to 28. An ISI score ≤ 7 indicates the *absence of insomnia*; 8–14 indicates *sub-threshold insomnia*; 15–21 *indicates moderate insomnia*; 22–28 *indicates severe insomnia* (Morin et al., 2011).

At baseline, 91 out of 146 participants (62.3%) reported using a mobile phone ≥ 4 h per day (LTMPU). Each participant with LTMPU provided written informed consent before undergoing magnetic resonance (MR) (within 2 weeks of completing the scale). Nine participants were excluded due to MRI motion artifacts. Finally, 82 participants with LTMPU were included.

### 2.2 MR imaging

All patients were examined using a 3.0 T whole-body scanner (Discovery MR750; GE Healthcare, Milwaukee, WI, USA) equipped with a 32-channel phased-array head coil. T2-weighted images (T2WI) acquisition parameters were as follows: TR = 5,613 ms, TE = 116 ms, slice thickness = 5.0 mm, slice spacing = 1.5 mm, and FOV = 26 cm. 3D T1-weighted imaging (T1WI) was acquired using a spoilt gradient echo sequence with a repetition time = 2.9 ms, echo time = 3.0 ms, inversion time = 450 ms, flip angle = 8°, slice thickness = 1 mm, matrix = 250 × 250, FOV = 22 cm × 22 cm.

### 2.3 Data preprocessing and EPVSs quantification

The image preprocessing encompasses multiple steps, as described below. Initially, N4 bias field corrections were implemented on both T1WI and T2WI images to eliminate magnetic field inhomogeneity. Subsequently, the grayscale values were standardised by normalising the intensities to fall within the range of [−1, 1] through clipping in the range of 0.1–99.9%. By employing a deep learning model (VB-Net) (Shi et al., 2022), which was embedded within an image analysis tool known as the uAI research portal (United Imaging Intelligence) (Shi et al., 2022; Wu et al., 2023), the skull was removed from the T1WI, and the entire brain was segmented into 109 regions of interest (ROIs) based on the DK atlas (Desikan et al., 2006).

These ROIs were grouped into 17 subregions (Supplementary Table 1) based on their anatomical proximity, functional homogeneity, and pathological relevance to EPVSs involvement. Key subregions include the basal ganglia, centrum semiovale, thalamus, and brainstem–established loci for glymphatic dysfunction (Wardlaw et al., 2020; Ineichen et al., 2022; van der Thiel et al., 2024). The final subregions included the bilateral frontal lobes, parietal lobes, occipital lobes, temporal lobes, basal ganglia, cerebellum, thalamus, centrum semiovale, and brainstem (Supplementary Table 1). Thereafter, the EPVSs lesions were automatically segmented from the T2WI images using a built-in VB-Net model (Zhang et al., 2024), demonstrating high accuracy for EPVSs segmentation with a recall and precision of 0.953 and 0.923, respectively (Zhang et al., 2024). The AI-generated masks were reviewed and modified by two experienced radiologists when necessary. Additionally, the T1WI and T2WI images were co-registered using a registration algorithm (Avants et al., 2014), transferring the segmentation mask from the T1WI space to the T2WI space. A comprehensive analysis was performed, computing 68 quantitative metrics of EPVSs lesions, including the number, volume, average length, and average curvature of the EPVSs lesions for each brain subregion.

### 2.4 Correlation analyses

To explore the association between the EPVSs metrics (namely, distributed regions, number, volume, mean length, and mean curvature) and human life status evaluated by the corresponding scales (e.g., MoCA scale for cognition, PSQI scale for sleep quality, ISI scale for insomnia, ESS scale for sleepiness, HAM-A scale for anxiety, and HAM-D scale for depression), relevant correlation analyses were conducted. The methods of correlation analysis were as follows: (1) for two measured values that conformed to a normal distribution, Pearson’s correlation analysis was employed; (2) when at least one of the two measured values did not conform to a normal distribution, Spearman’s correlation analysis was used. In multiple hypothesis testing, the false discovery rate (FDR) was utilised to correct the *p-value* of the correlation analysis, and an FDR-adjusted *p-value* < 0.05 was considered statistically significant. Correlation coefficient (*r*) represent the strength of correlation: r ≥ 0.90, very strong correlation; 0.70 ≤ r < 0.90, strong correlation; 0.40 ≤ r < 0.70, moderate correlation; 0.10 ≤ r < 0.40, weak correlation; r < 0.10, negligible correlation (Schober et al., 2018).

Based on the significant correlations identified, scatter plots were employed to illustrate the distribution of each pair of variables and their positive or negative associations. Scatter plots were constructed using RStudio (version 4.2.2). In addition, BrainNet Viewer (https://www.nitrc.org/projects/bnv/; Xia et al., 2013) emphasised the significant brain subregions in which the EPVSs metrics exhibited a significant correlation with clinical scale scores.

### 2.5 Partial correlation analyses

Considering the potential confounding effects of age and sex on EPVSs morphology and clinical status, partial correlation analyses were conducted using age and sex as covariates to assess the adjusted associations. Following the framework outlined in Section 2.4, normality testing was first applied to all the variables. Partial Pearson correlations were computed for pairs of variables satisfying normality assumptions; otherwise, partial Spearman correlations were used. The FDR method was rigorously applied to correct *p*-values across all comparisons, with statistical significance defined as FDR-adjusted *p* < 0.05.

### 2.6 Univariate and multivariate logistic regression analyses

Univariate and multivariate logistic regression analyses were conducted to identify robust characteristics correlated with clinical outcomes. A total of 70 features (68 EPVSs characteristics, age, and sex) were included in the univariate analysis for each of the six clinical statuses. Variables with *p* < 0.1 in univariate regression were advanced to multivariate regression, where statistical significance was defined as *p* < 0.05. Clinical scales were dichotomised as follows: a MoCA score ≥ 26 indicated *cognitive normalisation*, while a score < 26 indicated *cognitive impairment*; a PSQI score > 5 denoted *poor sleep quality*, and a score ≤ 5 denoted *good sleep quality*; an ISI score ≤ 7 was categorised as *non-insomnia*, and a score > 7 as *insomnia*; an ESS score ≤ 6 indicated *non-sleepiness*, while a score > 6 indicated *sleepiness*; a HAM-A score > 7 was considered *anxious*, and a score ≤ 7 was considered *non-anxious*; a HAM-D score > 7 was considered *depressed*, while a score ≤ 7 was considered *non-depressed*. Each clinical outcome was analysed separately.

## 3 Results

### 3.1 Participant characteristics

**Figure 1:**
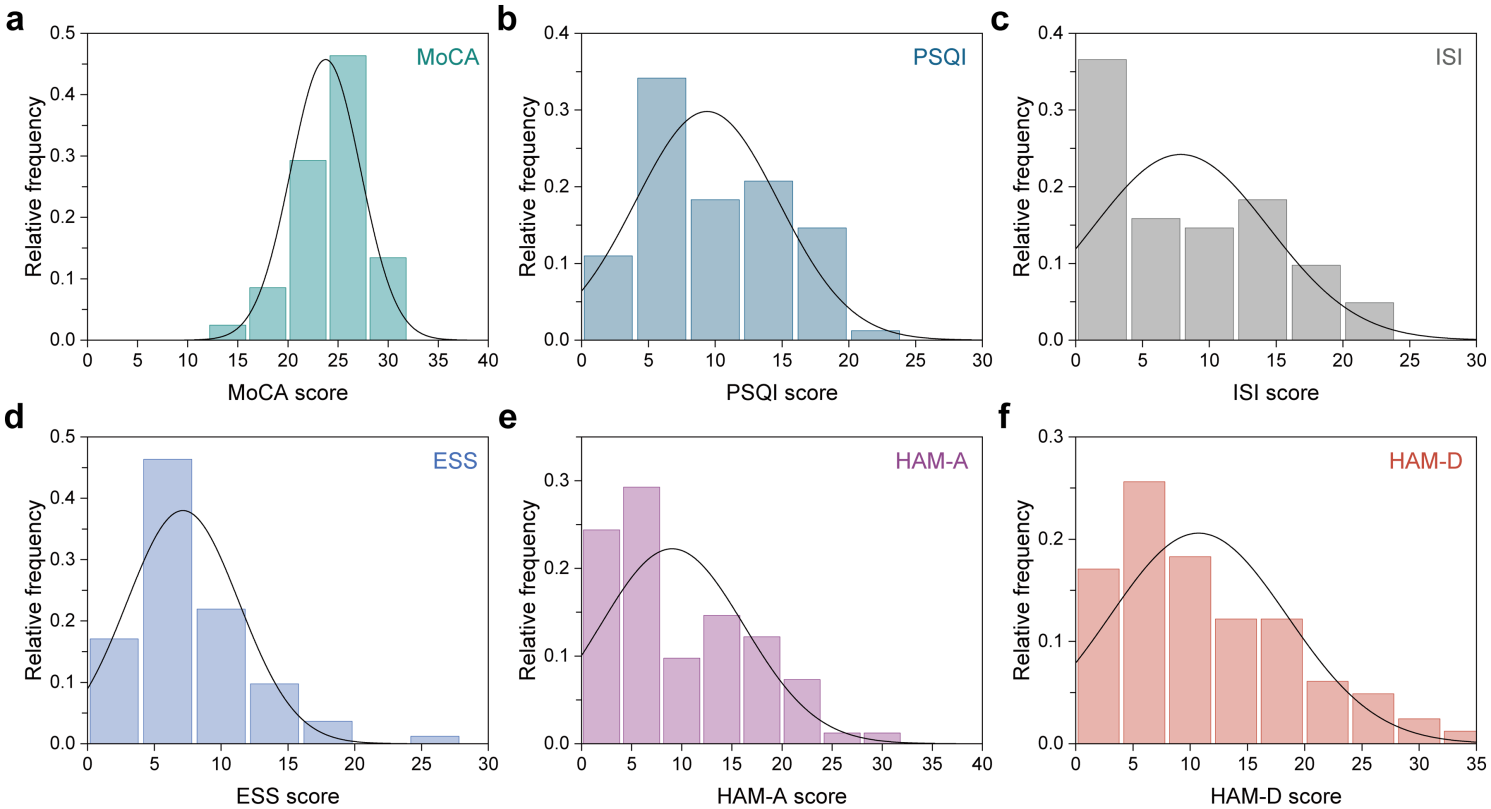
Distribution of six clinical scale scores across all participants. **(a)** MoCA, **(b)** PSQI, **(c)** ISI, **(d)** ESS, **(e)** HAM-A, and **(f)** HAM-D scale scores.

A total of 82 participants with LTMPU were retrospectively included. They underwent MR examinations and assessments using six clinical scales: the MoCA scale for cognition, PSQI scale for sleep quality, ISI scale for insomnia, ESS scale for sleepiness, HAM-A scale for anxiety, and HAM-D scale for depression. The demographic and clinical scale scores are presented in Table 1. The median age of all participants was 38.0 years, and 29.3% (24/82) were men. The distribution of clinical scale scores is plotted in Figure 1, with median scores of 25.0, 8.5, 7.0, 6.0, 6.0, and 9.0 for MoCA, PSQI, ISI, ESS, HAM-A, and HAM-D scale scores, respectively.

**Table 1:** Demographics of participants (total = 82).

Variables	Descriptive statistics	Disease (*n*, %)
Age (years)	38.0 (33.0, 43.0)	-
Sex (male, *n*, %)	24 (29.3%)	-
MoCA score	25.0 (21.8, 26.0)	Cognitive impairment (55, 67.1%)
PSQI score	8.5 (5.0, 14.3)	Poor sleep quality (54, 65.9%)
ISI score	7.0 (1.0, 14.0)	Insomnia (36, 43.9%)
ESS score	6.0 (4.0, 10.0)	Sleepiness (40, 48.8%)
HAM-A score	6.0 (3.8, 14.3)	Anxiety (37, 45.1%)
HAM-D score	9.0 (4.0, 16.0)	Depression (44, 53.7%)

### 3.2 Correlation analysis

**Figure 2:**
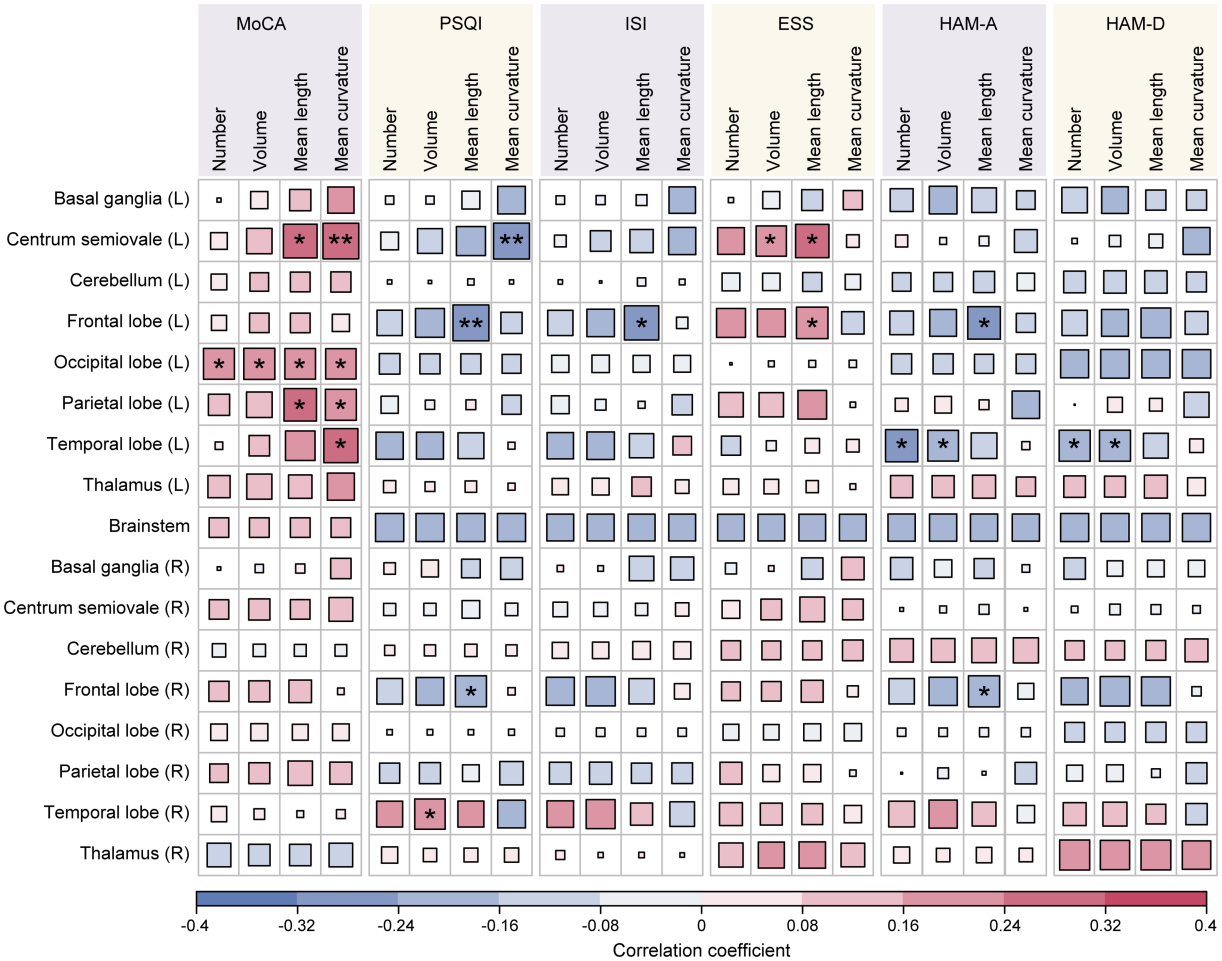
Correlation analyses between the 68 EPVSs characteristics and the 6 clinical scale scores. Asterisk represents two-tailed FDR-adjusted *p-value*, with * indicating *adjusted*-*p* < 0.05 and ** indicating *adjusted-p* < 0.01, showing that the correlations are statistically significant.

**Figure 3:**
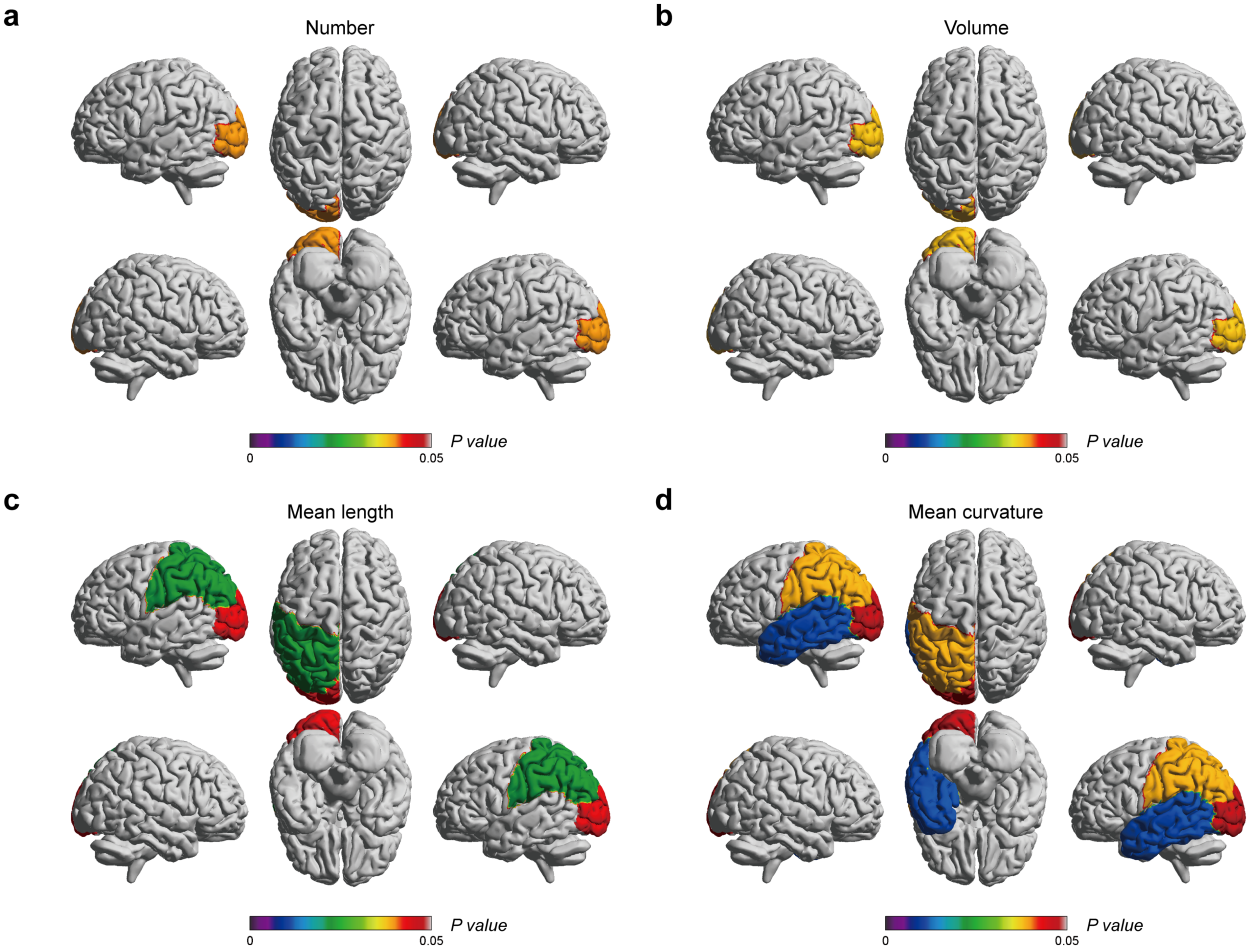
EPVSs characteristics that significantly correlate with MoCA scores, with subregions of the EPVSs distribution highlighted. **(a)** The number, **(b)** the volume, **(c)** the mean length, and **(d)** the mean curvature of EPVSs lesions in the corresponding subregions are correlated with MoCA scores. The centrum semiovale is not shown above. The scale bar represents the *adjusted p-value* in the correlation analyses, in which the *adjusted p-values* < 0.05 are highlighted.

Correlation analyses were carried out to determine the relationship between EPVSs characteristics (distributed sub-regions and quantitative indicators) and human living conditions evaluated by the six scales: the MoCA, PSQI, ISI, ESS, HAM-A, and HAM-D. As depicted in Figure 2, 23 pairs of indicators were significantly correlated after FDR correction, as shown in Supplementary Table 2. Specifically, the MoCA score was positively correlated with nine EPVSs features: the mean curvature of EPVSs lesions in the left centrum semiovale, left occipital lobe, left parietal lobe, and left temporal lobe; the mean length of EPVSs lesions in the left centrum semiovale, left occipital lobe, and left parietal lobe; and the number and volume of EPVSs lesions in the left occipital lobe. The subregions with EPVSs characteristics that were significantly correlated with the MoCA score are highlighted in Figure 3. The corresponding distributions of these nine pairs of EPVSs characteristics and MoCA scores are plotted in Supplementary Figure 1.

**Figure 4:**
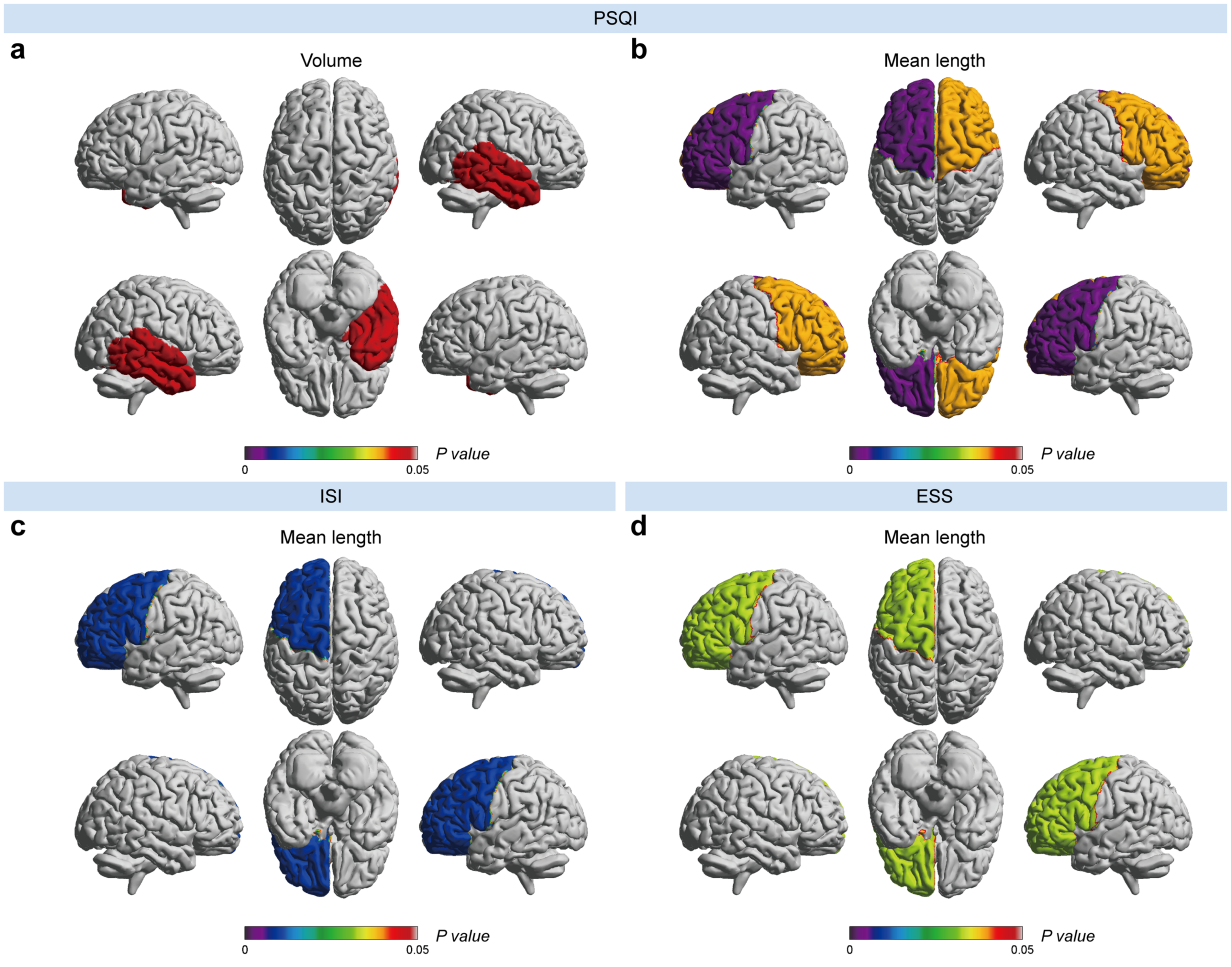
EPVSs characteristics that significantly correlate with sleep-related scores, with subregions of the EPVSs distribution highlighted. **(a)** The volume and **(b)** the mean length of EPVSs lesions in the corresponding subregions are correlated with PSQI scores. The mean length of EPVSs is significantly correlated with **(c)** ISI score and **(d)** ESS score. The centrum semiovale is not shown above. The scale bar represents the *adjusted p-value* in the correlation analyses, in which the *adjusted p-values* < 0.05 are highlighted.

In addition, the PSQI, ISI, and ESS were related to sleep assessment. Specifically, the PSQI score was positively correlated with the volume of the EPVSs in the right temporal lobe. At the same time, it exhibited a negative association with three EPVSs features: the mean curvature of the EPVSs in the left centrum semiovale and the mean length of the EPVSs in the left and right frontal lobes (Figures 4a,b). The ISI score was negatively correlated with the mean length of the EPVSs in the left frontal lobe (Figure 4c). In contrast, the ESS score was positively correlated with the mean length of the EPVSs in the left frontal lobe (Figure 4d), suggesting that the longer the EPVSs, the lower the ISI score and the less likely insomnia, whereas the higher the ESS score, the greater the degree of sleepiness. Moreover, the ESS score positively correlated with the EPVSs volume and mean length in the left centrum semiovale. The corresponding distributions of these EPVSs characteristics that were significantly correlated with the PSQI, ISI, and ESS scores are shown in Supplementary Figure 2.

**Figure 5:**
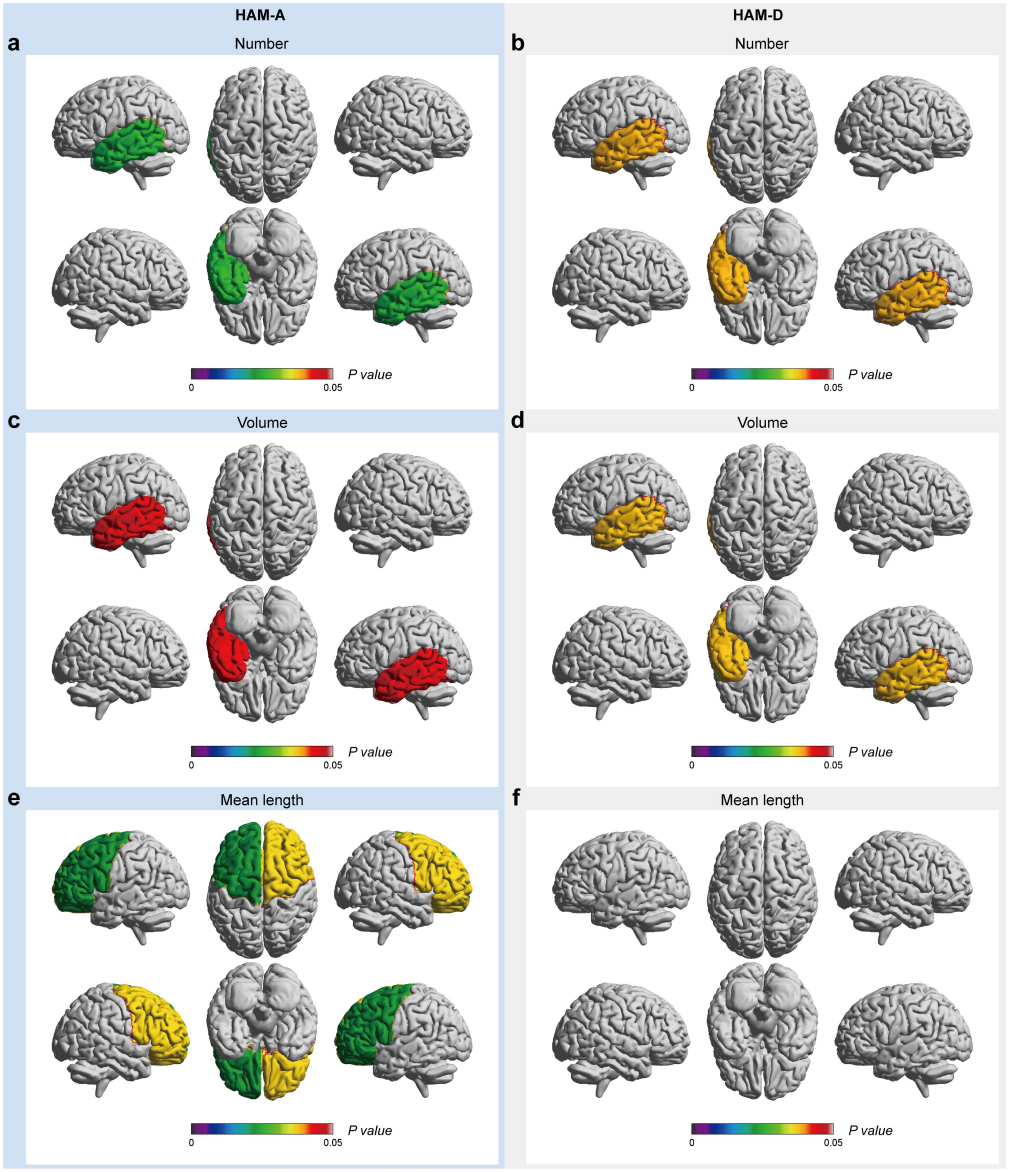
EPVSs characteristics that significantly correlate with mood-related scores, with subregions of the EPVSs distribution highlighted. The number **(a,b)**, the volume **(c,d)**, and the mean length **(e,f)** of EPVSs lesions in the corresponding subregions are significantly correlated with HAM-A and HAM-D scores. The scale bar represents the *adjusted p-value* in the correlation analyses, in which the *adjusted p-values* < 0.05 are highlighted.

In the mood-related correlation analyses, HAM-A and HAM-D scores were negatively correlated with the number and volume of EPVSs in the left temporal lobe (Figures 5a–d). Additionally, there were negative correlations between the HAM-A score and the mean length of the EPVSs in the left and right frontal lobes (Figure 5e). The corresponding distributions of the EPVSs characteristics that were significantly correlated with the HAM-A and HAM-D scores are shown in Supplementary Figure 3.

### 3.3 Partial correlation analysis

To account for potential confounding by age and sex, partial correlation analyses adjusted for these covariates were conducted across all 68 EPVSs features (17 subregions × 4 metrics: volume, number, mean length, and mean curvature) and six clinical scales. After FDR correction, six pairs of associations retain statistical significance (Table 2), while 17 previously identified correlations in unadjusted analyses (Supplementary Table 2) are attenuated below significance thresholds (FDR-adjusted *p* ≥ 0.05). Specifically, the MoCA scores were positively associated with EPVSs curvature in the left central semiovale and EPVSs length in the left parietal lobe. In contrast, the PSQI scores showed persistent negative correlations with EPVSs curvature in the left central semiovale and EPVSs length in the left frontal lobe. The bidirectional relationship between the left frontal EPVSs length and sleep phenotypes also survived adjustment; longer EPVSs were correlated with higher daytime sleepiness (ESS) but lower insomnia severity (ISI). These robust associations, unaffected by demographic confounders, highlight the independent role of EPVSs morphology in the sensorimotor, parietal, and frontal regions across cognitive and sleep-related outcomes. The full spatial patterns of the adjusted correlations are shown in Supplementary Figure 4.

**Table 2:** Pairs of EPVSs characteristics and clinical scale scores with significant correlations in age and sex-adjusted partial correlation analyses.

No.	Clinical scales	EPVSs characteristics	Coefficient	*Adjusted p-value*
1	MoCA	Mean_curvature_of_EPVSs_in_Left_centrum_semiovale	0.30	0.023
2	MoCA	Mean_length_of_EPVSs_in_Left_parietal_lobe	0.30	0.039
3	PSQI	Mean_curvature_of_EPVSs_in_Left_centrum_semiovale	−0.30	0.023
4	PSQI	Mean_length_of_EPVSs_in_Left_frontal_lobe	−0.31	0.028
5	ISI	Mean_length_of_EPVSs_in_Left_frontal_lobe	−0.28	0.035
6	ESS	Mean_length_of_EPVSs_in_Left_frontal_lobe	0.25	0.048

### 3.4 Univariate and multivariate logistic regression analysis

**Figure 6:**
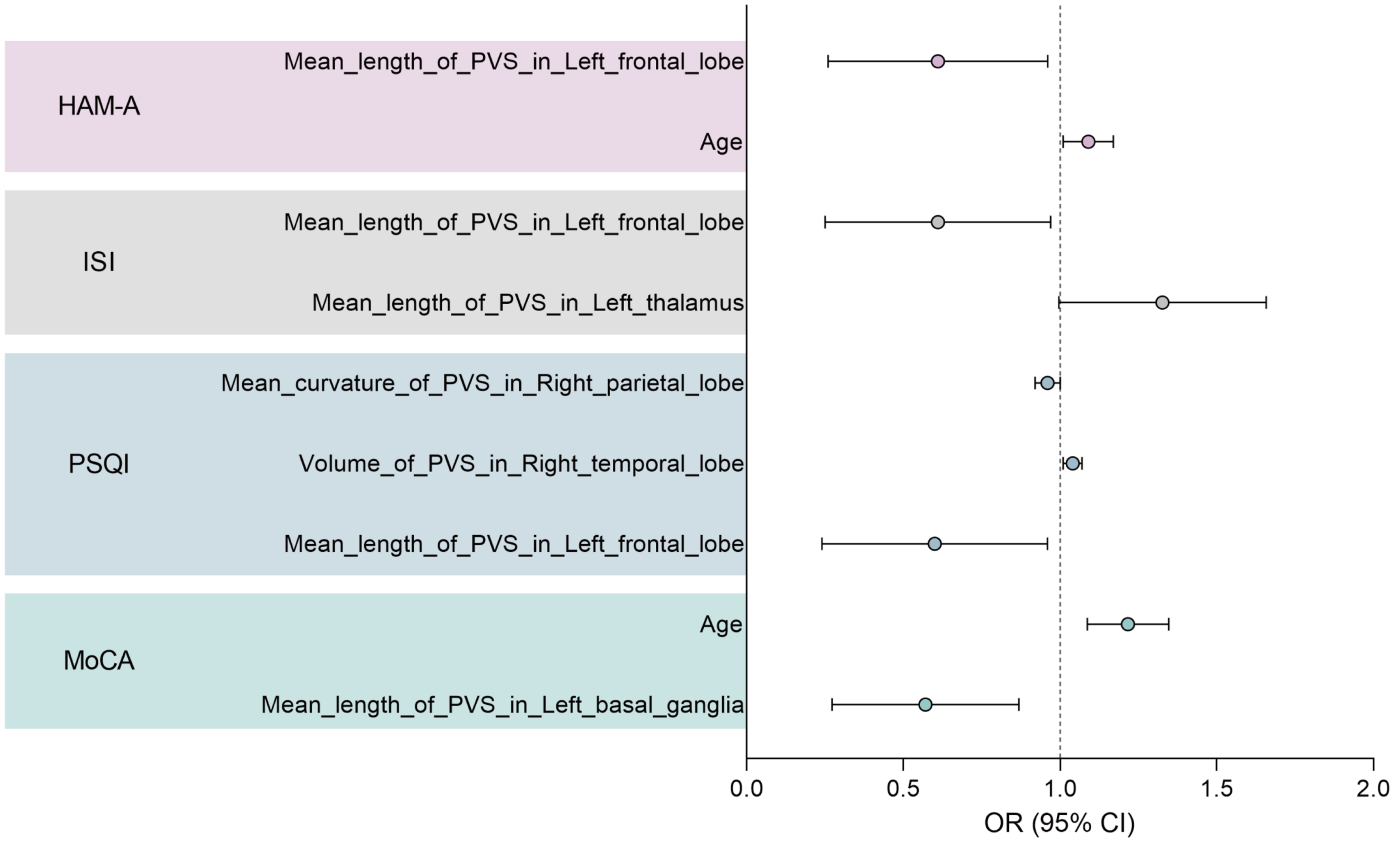
Forest plot showing significant variables with odds ratio (OR) in the multivariate logistic regression analysis.

Univariate and multivariate logistic regression analyses were conducted to identify further the robust characteristics significantly correlated with the clinical scale scores. The results are summarised in Supplementary Tables 3–8. All clinical scale scores were dichotomised to obtain disease and non-disease groups. Notably, no EPVSs features were found to be significant for sleepiness (ESS) or depression (HAM-D) in the multivariate regression analysis (*p* > 0.05). The forest plot in Figure 6 illustrates the significant variables with odds ratios (ORs) from multivariate logistic regression analyses. Age has emerged as a significant risk factor for anxiety (HAM-A) and cognitive impairment (MoCA). Specifically, for cognitive impairment, two characteristics were identified: the mean length of the EPVSs in the left basal ganglia and age. Additionally, the mean length of the EPVSs in the left frontal lobe has great potential for identifying poor sleep quality, insomnia, and anxiety.

## 4 Discussion

These non-invasive computational MRI-visible EPVSs metric approaches hold considerable promise for investigating different aspects of the glymphatic system. PVSs dysfunction is an emerging marker of sleep disturbance and neurodegenerative and neuroinflammatory diseases. However, little is known regarding MRI-visible EPVSs in the negative impact of MRI-visible EPVSs on young adults with LTMPU. In our study, computational MRI-visible EPVSs were associated with sleep disturbance (EDS), dementia, and mood disorders (anxiety) in young adults in the LTMPU. This understanding of computational MRI-visible EPVSs could provide novel insights into the pathologies underlying sleep disturbances, dementia, and mood disorders, which frequently co-occur and are mutually influenced. Our research further highlights that the association between MRI-visible EPVSs and measures such as HAM-A and ESS has not been extensively studied in the literature.

Our study found strong negative associations between the mean length of EPVSs in the left frontal lobe and HAM-A scores and between the mean curvature of EPVSs in the left temporal lobe and MoCA scores in young adults. It also demonstrated a positive association between the mean length of EPVSs in the left frontal lobe and the ESS. Accurate knowledge of the relevant brain areas for sleep disturbances (EDS), dementia, and mood disorders (anxiety) may provide a basis for prognostic and diagnostic tools.

We identified positive associations between the mean length of EPVSs in the left frontal lobe and ESS, suggesting a potential role for the frontal lobe in EDS, consistent with previous studies. This finding aligned with a previous review highlighting the involvement of specific brain regions, including the limbic system and the default mode network, in EDS (Pérez-Carbonell et al., 2022). Impaired glymphatic function, a marker of MRI-visible EPVSs, has been observed in rapid eye movement sleep behaviour disorders (Kenwood et al., 2022). We found a positive association between the mean length of EPVSs and ESS; however, to our knowledge, no study has assessed this relationship. Nevertheless, a recent study (Boucetta et al., 2017) has shown a negative correlation between ESS scores and cerebral blood flow in the medial prefrontal cortex. Another study that evaluated gray matter volume (GMV) showed that reduced GMV in the left ventromedial prefrontal cortex was significantly related to greater self-reported daytime sleepiness in the ESS (Si et al., 2022). Data on the role of perivascular spaces in healthy sleep and how their function may be impaired in human sleep disorders (Wardlaw et al., 2020). In addition, neuroimaging data are not invariably definitive, as variations in brain activity are contingent upon the behavioural impairments experienced by patients with sleepiness and the underlying cause of EDS (Pérez-Carbonell et al., 2022). Studies with larger sample sizes and wider age ranges are required to confirm this association.

In primates, the prefrontal cortex is pivotal in regulating anxiety (Kenwood et al., 2022). The results of our study underscore this relationship, demonstrating robust negative correlations between the mean length of EPVSs in the left frontal lobe and HAM-A scores. On MRI assessment, youths with anxiety disorders compared to healthy subjects had decreased gray matter volumes in the inferior frontal gyrus (ventrolateral prefrontal cortex) (Strawn et al., 2015). A systematic review and meta-analysis also provided evidence of hypoconnectivity between the amygdala and medial frontal gyrus, anterior cingulate cortex, and cingulate gyrus in patients with anxiety disorders (Zugman et al., 2023). However, to the best of our knowledge, no previous study assessed the association between anxiety and MRI-visible EPVSs. Mood disorders, anxiety, and depression are associated with elevated levels of inflammation (Guo et al., 2023). Perivascular spaces play a critical role in maintaining homeostasis and priming neuroinflammation (Ineichen et al., 2022).

Furthermore, there is a relationship between EPVSs, neuroinflammation, and blood–brain barrier function in neuromyelitis optical spectrum disorder (Yao et al., 2022). Our understanding of the pathophysiology of anxiety and the role of perivascular spaces has made significant strides. However, many critical gaps in our knowledge still require further exploration (Craske and Stein, 2016; Wardlaw et al., 2020). Our findings may improve our understanding of anxiety and lead to promising yet uncharted therapeutic territory.

Our study’s strong negative associations between the mean curvature of the EPVSs in the left temporal lobe and MoCA agree with previous studies. High-resolution MRI has enabled the correlation of perivascular space morphology with a spectrum of physiological and pathological states, including cognitive function, vascular risk factors, cerebrovascular and neurodegenerative brain lesions, sleep patterns, and cerebral haemodynamics (Wardlaw et al., 2020). Jeong et al. (2022) suggested that the centrum semiovale (EPVSs) is associated with the progression of cognitive decline in an amyloid-independent manner (Jeong et al., 2022). Previous studies have reported changes in EPVSs-related MRI parameters in patients with mild cognitive impairment and Alzheimer’s disease (AD) (Kamagata et al., 2022). The same study also showed that the EPVSs burden in the centrum semiovale may be a risk marker for early cognitive impairment (Pase et al., 2023). Atrophy of the medial temporal lobe or hippocampus, as observed on MRI, is the most well-established neurodegenerative biomarker of AD (Jack et al., 2004). The glymphatic system is an exciting new target for AD (Harrison et al., 2020). Therefore, we speculate that temporally non-invasive computational MRI-visible EPVSs metrics may help predict the risk of syndromal conversion in early AD.

As discussed previously, while this study is pioneering in the metrics derived from this computational EPVSs segmentation, the limitations of this study include (a) the need for longitudinal studies and (b) larger sample sizes and multi-center ranges. An additional limitation of the study design was the lack of comparison between EPVSs computational metrics and EPVSs visual ratings. Further research with expanded cohorts and multicentre approaches is required to substantiate the reliability and generalisability of our model.

## 5 Conclusion

The metrics derived from computational EPVSs segmentation provide valuable insights into the pathophysiology of MRI-visible EPVSs. The EPVSs indicate impairment of normal brain fluid, waste clearance, microvascular dysfunction, and impaired glymphatic exchange. These are relevant for understanding the brain fluid dynamics underlying mood disorders, sleep disturbances, and cognitive impairment in young adults, particularly concerning EDS and anxiety. It is anticipated that emerging techniques and imaging biomarkers will soon be integrated into clinical practice to enhance diagnostic accuracy and inform therapeutic strategies. Newer techniques and imaging markers will soon be translated into clinical practice to support clinical diagnostics and therapeutic interventions.

## Data Availability

The raw data supporting the conclusions of this article will be made available by the authors, without undue reservation.
