# Supplementary material for: MRI-based machine learning analysis of perivascular spaces and their link to sleep disturbances, dementia, and mental distress in young adults with long-time mobile phone use
Source: Front Neurosci. 2025 Apr 28;19:1555054. doi: 10.3389/fnins.2025.1555054 (PMC12066657; doi:10.3389/fnins.2025.1555054)
Supplement: Supplementary file 1 [file Data_Sheet_1.docx]

**Supplementary Table 1. Consolidation of 109 DK atlas ROIs into 17 brain subregions for EPVSs analysis.** Anatomical structures such as Cerebral_WM, Corpus_Callosum, and Cingulate_Gyrus were categorized into frontal, parietal, occipital, and temporal lobes based on their proximity principle.

| No. | Brain subregions | Anatomical structures included | Rationale for merging |
| --- | --- | --- | --- |
| 1 | Frontal_Lobe_L | Precentral_L, Frontal_Sup_L, Frontal_Mid_Rostral_L, Frontal_Mid_Caudal_L, Frontalpole_L, Orbitofrontal_Lat_L, Orbitofrontal_Med_L, Parsopercularis_L, Parsorbitalis_L, Parstriangularis_L | Grouped bilaterally for motor and executive function analysis. Adjacent structures merged by spatial proximity |
| 2 | Frontal_Lobe_R | Precentral_R, Frontal_Sup_R, Frontal_Mid_Rostral_R, Frontal_Mid_Caudal_R, Frontalpole_R, Orbitofrontal_Lat_R, Orbitofrontal_Med_R, Parsopercularis_R, Parsorbitalis_R, Parstriangularis_R | Grouped bilaterally for symmetry and analytical efficiency |
| 3 | Parietal_Lobe_L | Postcentral_L, Paracentral_L, Parietal_Sup_L, Parietal_Inf_L, Precuneus_L, Supramarginal_L | Functional homogeneity (sensory integration and spatial processing) and anatomical adjacency |
| 4 | Parietal_Lobe_R | Postcentral_R, Paracentral_R, Parietal_Sup_R, Parietal_Inf_R, Precuneus_R, Supramarginal_R | Grouped bilaterally to maintain functional symmetry and reduce dimensionality |
| 5 | Occipital_Lobe_L | Cuneus_L, Lingual_L, Pericalcarine_L, Occipital_Lat_L | Merged for visual processing coherence. Adjacent white matter included based on proximity |
| 6 | Occipital_Lobe_R | Cuneus_R, Lingual_R, Pericalcarine_R, Occipital_Lat_R | Grouped bilaterally to analyze hemispheric differences in visual pathway EPVSs distribution |
| 7 | Temporal_Lobe_L | Hippocampus_L, Parahippocampal_L, Entorhinal_L, Fusiform_L, Temporal_Sup_L, Temporal_Mid_L, Temporal_Inf_L, Temporalpole_L, Temporal_Sup_Banks_L, Transversetemporal_L | Merged to assess EPVSs in memory-related structures |
| 8 | Temporal_Lobe_R | Hippocampus_R, Parahippocampal_R, Entorhinal_R, Fusiform_R, Temporal_Sup_R, Temporal_Mid_R, Temporal_Inf_R, Temporalpole_R, Temporal_Sup_Banks_R, Transversetemporal_R | Grouped bilaterally for symmetry and functional homogeneity (auditory and memory processing) |
| 9 | Cerebellum_L | Cerebellum_Cortex_L, Cerebellum_WM_L | Analyzed separately to evaluate posterior fossa EPVSs patterns distinct from supratentorial regions |
| 10 | Cerebellum_R | Cerebellum_Cortex_R, Cerebellum_WM_R | Grouped bilaterally to assess cerebellar hemispheric symmetry in EPVSs distribution |
| 11 | Thalamus_L | Thalamus_L, VentralDC_L | Merged due to shared involvement in glymphatic drainage dysfunction |
| 12 | Thalamus_R | Thalamus_R, VentralDC_R | Grouped bilaterally for consistency with thalamocortical connectivity analysis |
| 13 | Basal_Ganglia_L | Amygdala_L, Caudate_L, Putamen_L, Pallidum_L, Accumbens_Area_L, Insula_L | High EPVSs density in caudate/putamen linked to CSVD pathology. Insula included for neuroinflammatory roles. |
| 14 | Basal_Ganglia_R | Amygdala_R, Caudate_R, Putamen_R, Pallidum_R, Accumbens_Area_R, Insula_R | Grouped bilaterally to analyze EPVSs asymmetry in CSVD biomarkers |
| 15 | Centrum_semiovale_L | Centrum_semiovale_L | Key region for assessing white matter EPVSs reflecting glymphatic dysfunction |
| 16 | Centrum_semiovale_R | Centrum_semiovale_R | Grouped bilaterally to study hemispheric differences in fluid clearance efficiency |
| 17 | Brainstem | Pons, Midbrain, Medulla, SCP | Critical for perivascular fluid dynamics and neuroinflammatory processes |

**Supplementary Table 2. Pairs of EPVSs characteristics and clinical scale scores with significant correlations in correlation analyses.**

| No. | Clinical scales | EPVSs characteristics | Coefficient | *Adjusted p-value* |
| --- | --- | --- | --- | --- |
| 1 | MoCA | Mean_curvature_of_EPVSs_in_Left_centrum_semiovale | 0.30 | 0.006 |
| 2 | MoCA | Mean_curvature_of_EPVSs_in_Left_occipital_lobe | 0.22 | 0.047 |
| 3 | MoCA | Mean_curvature_of_EPVSs_in_Left_parietal_lobe | 0.23 | 0.039 |
| 4 | MoCA | Mean_curvature_of_EPVSs_in_Left_temporal_lobe | 0.28 | 0.012 |
| 5 | PSQI | Mean_curvature_of_EPVSs_in_Left_centrum_semiovale | -0.31 | 0.005 |
| 6 | MoCA | Mean_length_of_EPVSs_in_Left_centrum_semiovale | 0.27 | 0.014 |
| 7 | MoCA | Mean_length_of_EPVS_in_Left_occipital_lobe | 0.22 | 0.043 |
| 8 | MoCA | Mean_length_of_EPVSs_in_Left_parietal_lobe | 0.25 | 0.022 |
| 9 | PSQI | Mean_length_of_EPVSs_in_Left_frontal_lobe | -0.31 | 0.005 |
| 10 | PSQI | Mean_length_of_EPVSs_in_Right_frontal_lobe | -0.23 | 0.039 |
| 11 | ISI | Mean_length_of_EPVSs_in_Left_frontal_lobe | -0.28 | 0.011 |
| 12 | ESS | Mean_length_of_EPVSs_in_Left_centrum_semiovale | 0.26 | 0.016 |
| 13 | ESS | Mean_length_of_EPVSs_in_Left_frontal_lobe | 0.24 | 0.033 |
| 14 | HAM-A | Mean_length_of_EPVSs_in_Left_frontal_lobe | -0.25 | 0.022 |
| 15 | HAM-A | Mean_length_of_EPVSs_in_Right_frontal_lobe | -0.23 | 0.037 |
| 16 | MoCA | Number_of_EPVSs_in_Left_occipital_lobe | 0.23 | 0.040 |
| 17 | HAM-A | Number_of_EPVSs_in_Left_temporal_lobe | -0.25 | 0.026 |
| 18 | HAM-D | Number_of_EPVSs_in_Left_temporal_lobe | -0.23 | 0.039 |
| 19 | MoCA | Volume_of_EPVSs_in_Left_occipital_lobe | 0.23 | 0.038 |
| 20 | PSQI | Volume_of_EPVSs_in_Right_temporal_lobe | 0.22 | 0.048 |
| 21 | ESS | Volume_of_EPVSs_in_Left_centrum_semiovale | 0.22 | 0.049 |
| 22 | HAM-A | Volume_of_EPVSs_in_Left_temporal_lobe | -0.22 | 0.043 |
| 23 | HAM-D | Volume_of_EPVSs_in_Left_temporal_lobe | -0.23 | 0.038 |


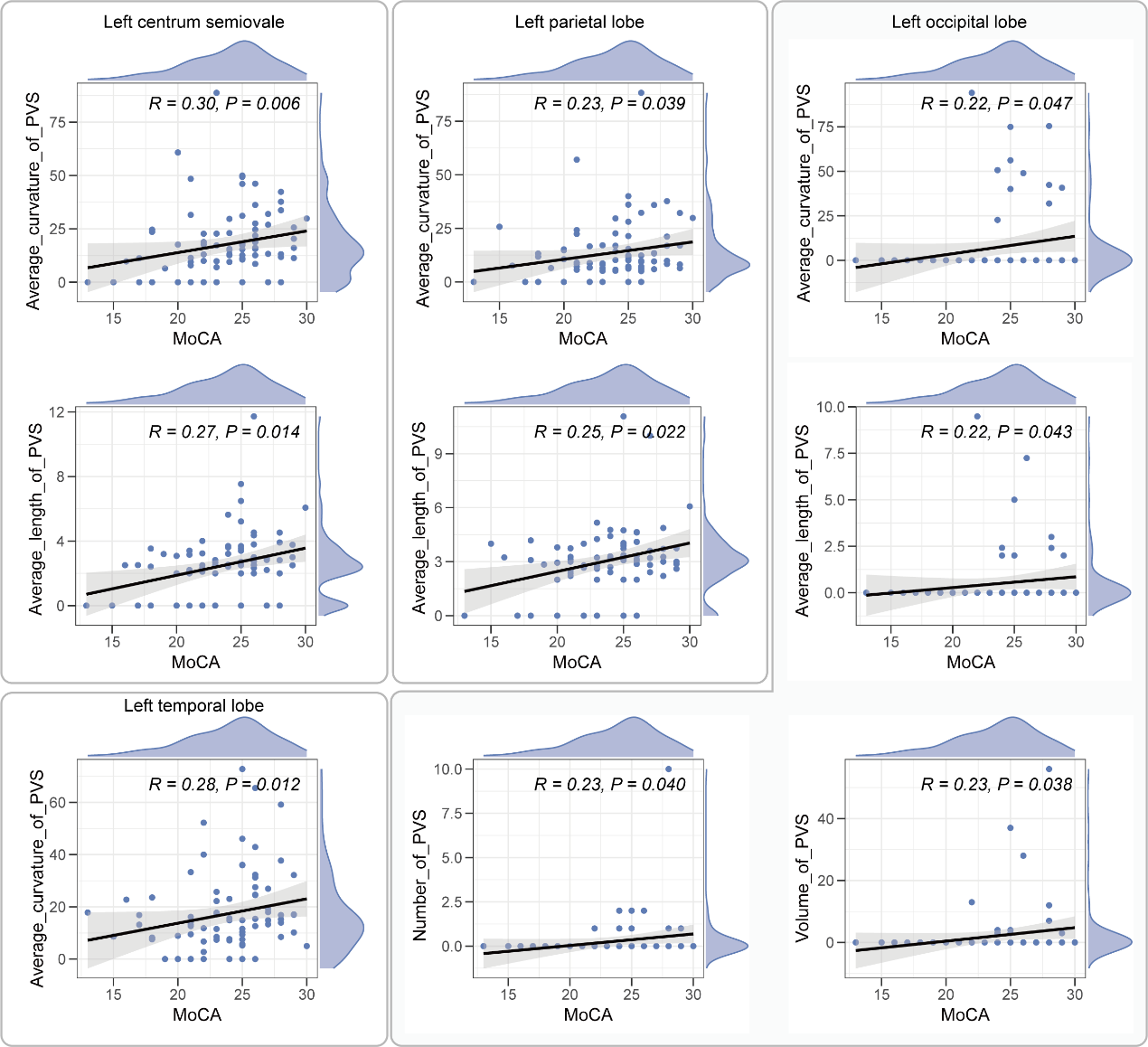


**Supplementary Figure 1. Distribution of EPVSs features significantly associated with MoCA scores.**


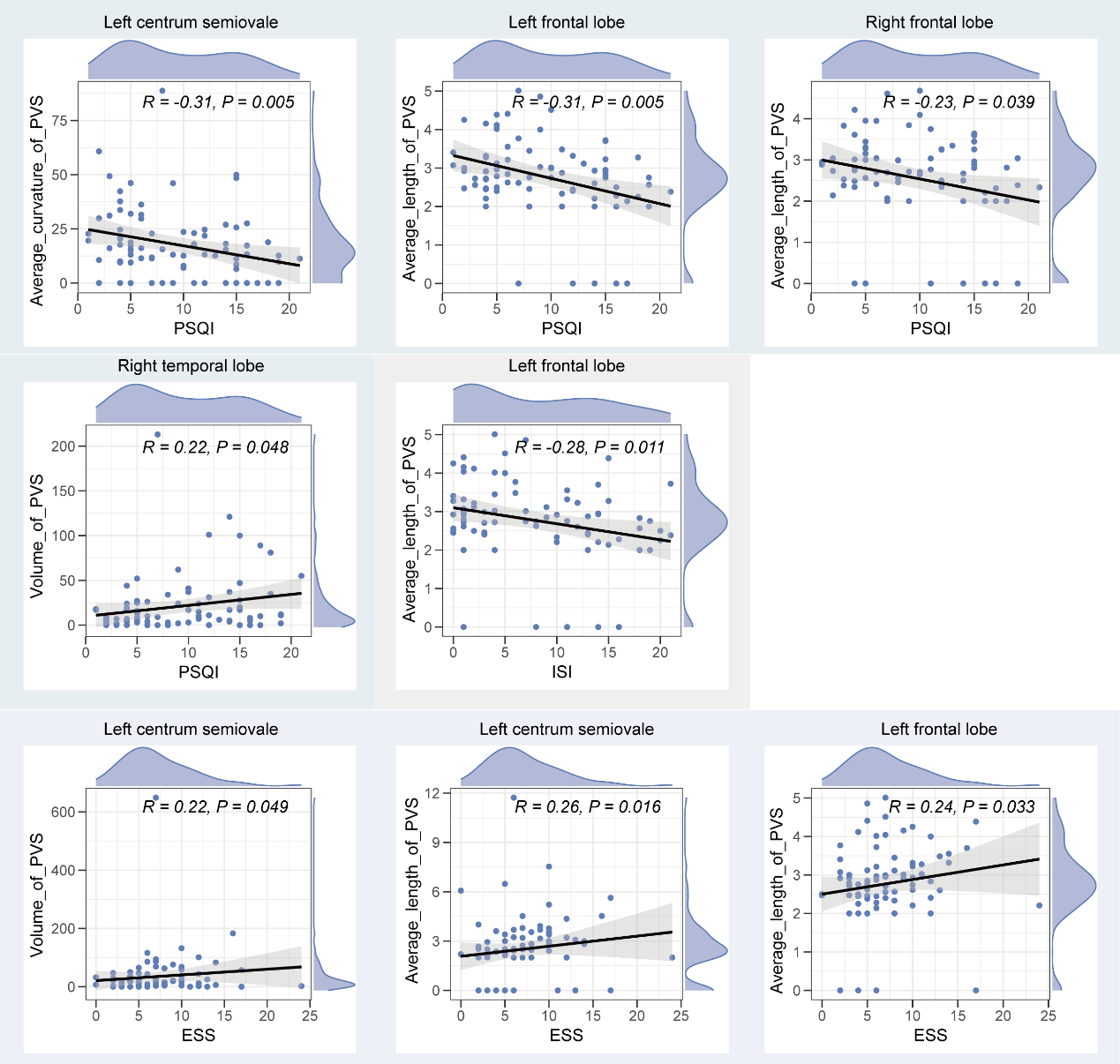


**Supplementary Figure 2. Distribution of EPVSs features significantly associated with three scales related to sleep assessment.**


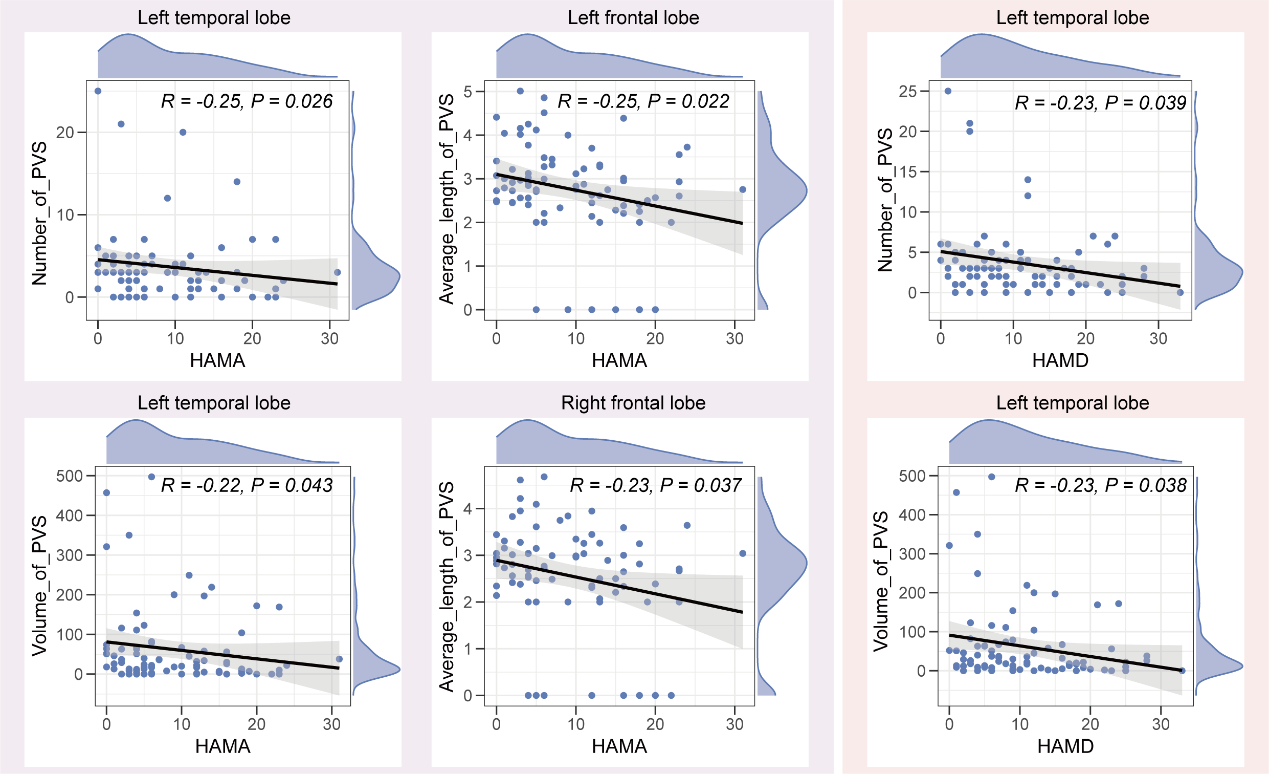


**Supplementary Figure 3. Distribution of E features significantly associated with HAM-A and HAM-D scores.**


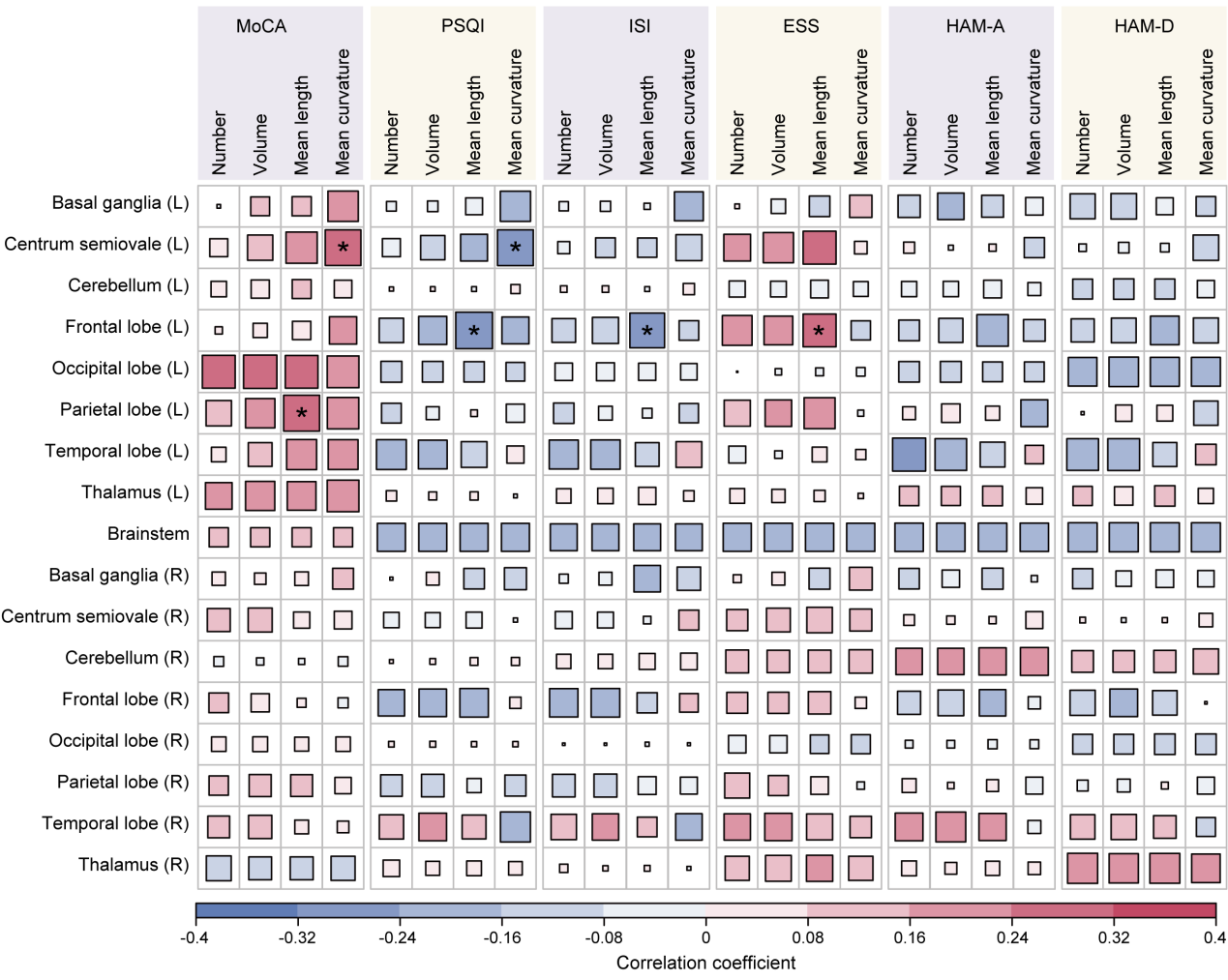


**Supplementary Figure 4. Partial correlation analyses between the 68 EPVSs characteristics and the 6 clinical scale scores.** Asterisk represents two-tailed FDR *adjusted p-value*, with * indicating *adjusted*-*p* < 0.05, showing that the correlations are statistically significant after adjusting for age and sex.

**Supplementary Table 3. Univariate and multivariate logistic regression analysis to identify risk factors for cognitive impairment (based on the MoCA scale).**

| Variables |  | Univariate |  |  | Multivariate |  |
| --- | --- | --- | --- | --- | --- | --- |
|  | Coefficient | OR (95% CI) | *P* | Coefficient | OR (95% CI) | *P* |
| Volume_of_EPVSs_in_Right_centrum_semiovale | -0.01 | 0.99 (0.98 - 1.00) | 0.099 | - | - | - |
| Mean_curvature_of_EPVSs_in_Left_cerebellum | -0.02 | 0.98 (0.96 - 1.00) | 0.098 | - | - | - |
| Mean_curvature_of_EPVSs_in_Left_thalamus | -0.01 | 0.99 (0.97 - 1.00) | 0.093 | - | - | - |
| Mean_length_of_EPVSs_in_Right_frontal_lobe | -0.43 | 0.65 (0.40 - 1.07) | 0.088 | - | - | - |
| Mean_length_of_EPVSs_in_Left_centrum_semiovale | -0.23 | 0.79 (0.61 - 1.03) | 0.080 | - | - | - |
| Volume_of_EPVSs_in_Left_basal_ganglia | -0.01 | 0.99 (0.99 - 1.00) | 0.076 | - | - | - |
| Mean_length_of_EPVSs_in_Left_basal_ganglia | -0.38 | 0.68 (0.47 - 0.98) | 0.039 | -0.66 | 0.52 (0.30 - 0.89) | 0.018 |
| Mean_length_of_EPVSs_in_Left_temporal_lobe | -0.20 | 0.82 (0.68 - 0.99) | 0.038 | - | - | - |
| Mean_curvature_of_EPVSs_in_Left_temporal_lobe | -0.04 | 0.96 (0.93 - 0.99) | 0.016 | - | - | - |
| Age | 0.13 | 1.14 (1.06 - 1.24) | 0.001 | 0.19 | 1.21 (1.09 - 1.35) | 0.001 |

**Supplementary Table 4. Univariate and multivariate logistic regression analysis to identify risk factors for poor sleep quality (based on the PSQI scale).**

| Variables |  | Univariate |  |  | Multivariate |  |
| --- | --- | --- | --- | --- | --- | --- |
|  | Coefficient | OR (95% CI) | *P* | Coefficient | OR (95% CI) | *P* |
| Mean_length_of_EPVSs_in_Left_frontal_lobe | -0.45 | 0.64 (0.38 - 1.06) | 0.084 | -0.64 | 0.53 (0.28 - 0.99) | 0.045 |
| Volume_of_EPVSs_in_Right_temporal_lobe | 0.02 | 1.02 (1.00 - 1.05) | 0.081 | 0.04 | 1.04 (1.01 - 1.07) | 0.020 |
| Mean_curvature_of_EPVSs_in_Right_parietal_lobe | -0.03 | 0.97 (0.94 - 1.00) | 0.076 | -0.04 | 0.96 (0.92 - 1.00) | 0.036 |

**Supplementary Table 5. Univariate and multivariate logistic regression analysis to identify risk factors for insomnia (based on the ISI scale).**

| Variables |  | Univariate |  |  | Multivariate |  |
| --- | --- | --- | --- | --- | --- | --- |
|  | Coefficient | OR (95% CI) | *P* | Coefficient | OR (95% CI) | *P* |
| Mean_length_of_EPVSs_in_Right_frontal_lobe | -0.35 | 0.71 (0.47 - 1.06) | 0.097 | - | - | - |
| Volume_of_EPVSs_in_Right_basal_ganglia | -0.01 | 0.99 (0.99 - 1.00) | 0.096 | - | - | - |
| Mean_length_of_EPVSs_in_Left_thalamus | 0.19 | 1.21 (0.97 - 1.50) | 0.085 | 0.26 | 1.30 (1.01 - 1.67) | 0.042 |
| Mean_length_of_EPVSs_in_Left_frontal_lobe | -0.64 | 0.53 (0.31 - 0.88) | 0.014 | -0.63 | 0.54 (0.29 - 1.00) | 0.049 |

**Supplementary Table 6. Univariate and multivariate logistic regression analysis to identify risk factors for sleepiness (based on the ESS scale).** No significant results were observed after multivariate analysis (*P* > 0.05).

| Variables |  | Univariate |  |
| --- | --- | --- | --- |
|  | Coefficient | OR (95% CI) | *P* |
| Number_of_EPVSs_in_Left_frontal_lobe | 0.06 | 1.06 (0.99 - 1.14) | 0.093 |
| Mean_length_of_EPVSs_in_Right_frontal_lobe | 0.38 | 1.46 (0.95 - 2.23) | 0.081 |
| Mean_length_of_EPVSs_in_Left_centrum_semiovale | 0.23 | 1.26 (0.97 - 1.64) | 0.080 |
| Number_of_EPVSs_in_Left_centrum_semiovale | 0.14 | 1.15 (0.99 - 1.34) | 0.063 |
| Volume_of_EPVSs_in_Left_centrum_semiovale | 0.02 | 1.02 (1.00 - 1.03) | 0.038 |
| Mean_length_of_EPVSs_in_Left_frontal_lobe | 0.53 | 1.70 (1.04 - 2.79) | 0.034 |

**Supplementary Table 7. Univariate and multivariate logistic regression analysis to identify risk factors for anxiety (based on the HAM-A scale).**

| Variables |  | Univariate |  |  | Multivariate |  |
| --- | --- | --- | --- | --- | --- | --- |
|  | Coefficient | OR (95% CI) | *P* | Coefficient | OR (95% CI) | *P* |
| Mean_curvature_of_EPVSs_in_Left_frontal_lobe | -0.08 | 0.92 (0.84 - 1.01) | 0.075 | - | - | - |
| Sex | -0.96 | 0.38 (0.14 - 1.07) | 0.066 | - | - | - |
| Age | 0.07 | 1.07 (1.00 - 1.14) | 0.053 | 0.08 | 1.09 (1.01 - 1.17) | 0.032 |
| Mean_length_of_EPVSs_in_Left_frontal_lobe | -0.65 | 0.52 (0.31 - 0.88) | 0.014 | -0.61 | 0.54 (0.30 - 0.99) | 0.046 |

**Supplementary Table 8. Univariate and multivariate logistic regression analysis to identify risk factors for depression (based on the HAM-D scale).** No significant results were observed after multivariate analysis (*P* > 0.05).

| Variables |  | Univariate |  |
| --- | --- | --- | --- |
|  | Coefficient | OR (95% CI) | *P* |
| Number_of_EPVSs_in_Left_temporal_lobe | -0.11 | 0.90 (0.79 - 1.02) | 0.096 |
| Mean_length_of_EPVSs_in_Left_frontal_lobe | -0.41 | 0.67 (0.42 - 1.06) | 0.088 |
| Mean_curvature_of_EPVSs_in_Left_parietal_lobe | -0.03 | 0.97 (0.93 - 1.00) | 0.085 |
| Volume_of_EPVSs_in_Left_temporal_lobe | -0.01 | 1.00 (0.99 - 1.01) | 0.081 |
| Age | 0.06 | 1.06 (0.99 - 1.13) | 0.073 |
